# Identification of amyloid plaques in the brain using an x-ray photon-counting strip detector

**DOI:** 10.1371/journal.pone.0228720

**Published:** 2020-02-11

**Authors:** Bahaa Ghammraoui, Aldo Badano

**Affiliations:** Division of Imaging, Diagnostics and Software Reliability, Office of Science and Engineering Laboratories, CDRH/FDA, Silver Spring, Maryland, United States of America; Massaschusetts General Hospital and Harvard Medical School, UNITED STATES

## Abstract

Brain aggregates of *β* amyloid (*β*A) protein plaques have been widely recognized as associated with many neurodegenerative diseases, and their identification can assist in the early diagnosis of Alzheimer’s disease. We investigate the feasibility of using a spectral x-ray coherent scatter system with a silicon strip photon-counting detector for identifying brain *β*A protein plaques. This approach is based on differences in the structure of amyloid, white and grey matter in the brain. We simulated an energy- and angular-dispersive X-ray diffraction system with an x-ray pencil beam and Silicon strip sensor, energy-resolving detectors. The polychromatic beam is geometrically focused toward a region of interest in the brain. First, the open-source MC-GPU code for Monte Carlo transport was modified to accommodate the detector model. Second, brain phantoms with and without *β*A were simulated to assess the method and determine the radiation dose required to obtain acceptable statistical power. For *β*A targets of 3, 4 and 5 mm sizes in a 15-cm brain model, the required incident exposure was about 0.44 mR from a 60 kVp tungsten spectrum and 3.5 mm of added aluminum filtration. The results suggest that the proposed x-ray coherent scatter technique enables the use of high energy x-ray spectra and therefore has the potential to be used for accurate in vivo detection and quantification of *β*A in the brain within acceptable radiation dose levels.

## Introduction

Clinical identification of Alzheimer’s disease has relied primarily on the evaluation of the patient’s cognitive symptoms. Amyloid *β* (*β*A) positron emission tomography (PET) imaging uses radioactive tracers to highlight amyloid plaques in the brain [1] but its use has been limited, as it is time consuming and expensive due to the injection of short-lived radioisotopes. X-ray phase contrast imaging methods have shown potential for imaging dense amyloid plaques with high resolution [2, 3]. However, x-ray phase contrast is not currently available clinically since it requires sophisticated instrumentation including a synchrotron or microfocus x-ray source, x-ray optics, and high resolution x-ray detectors. In addition, the mechanism by which *β*A offers additional x-ray phase contrast has not been unequivocally described.

Several x-ray powder diffraction studies have been conducted on brain *β*A to determine their x-ray diffraction characteristics [4]. These studies showed that two major peaks are present in the *β*A x-ray coherent scatter profile at momentum transfer corresponding to 0.55 nm^−1^ and 1.064 nm^−1^. Because of this characteristic property, coherent scatter methods have the potential to enable brain amyloid characterization without the need for radioactive materials. Dahal *et al*. [5, 6] demonstrated the potential of the coherent scatter tomography imaging method to characterize aggregates based on their molecular structures and as a non-invasive method to identify amyloid aggregates in the human head. Although coherent scatter computed tomography is a very promising imaging modality, its use has been limited by the need to translate and rotate the pencil beam geometry, resulting in increased scan time and radiation dose to the patient [7].

In this study, we investigated the use of a multiple-angle, energy-dispersive coherent scatter system that makes use of a narrowly collimated x-ray beam directed at suspicious areas inside the brain. The system used silicon energy-resolving photon-counting strip detectors with edge-on geometry [8, 9] geometrically focused toward the suspicious area being interrogated. Focused silicon strip detectors measure all scattered photons coming exclusively from the suspicious region in the brain. In addition, the energy-resolving ability of the silicon detector allows for different energy ranges within a single scan, resulting in improved counting statistics and more efficient scatter profile restoration. As a first step in exploring this approach, we used computer simulations with digital human brain phantom to evaluate different aspects of the proposed technique.

## Materials and methods

### Simulation setup

To simulate the coherent scatter system, we used a modified version of MC-GPU [10], a Graphical Processing Unit version of PENELOPE, the Monte Carlo particle transport simulation code, that incorporates an improved model of x-ray coherent scattering using experimentally measured molecular interference functions [11].

**Fig 1:**
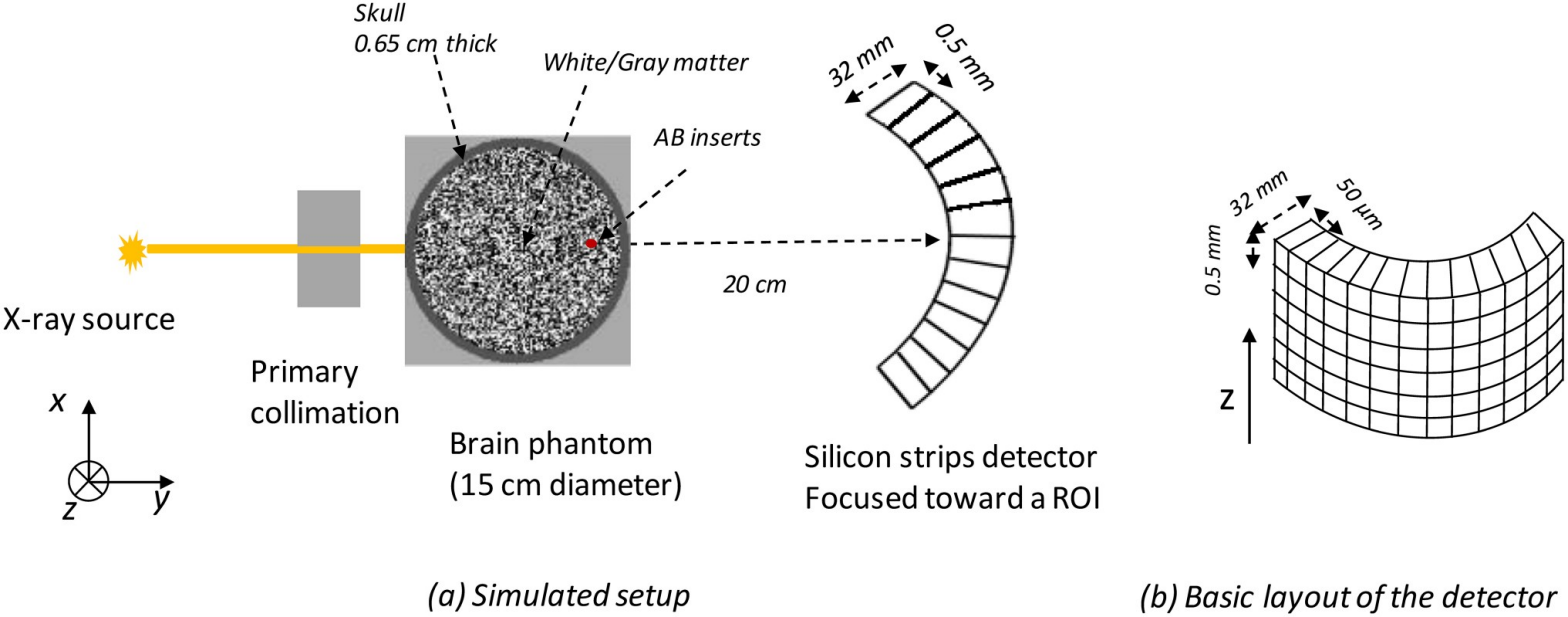
Schematic representation of the coherent scatter system combining energy-resolved information at multiple scattering angles with a polychromatic pencil beam and an energy-resolved detector.

The simulation setup is sketched in Fig 1 and includes a collimated x-ray rectangular beam source (0.5×0.5 mm^2^) aimed at the center of the suspected region in the brain. We use a 60 kV tungsten anode source filtered by 3.5 mm of aluminum and collecting all the scattered radiation at small angles (< 10°) with a 2D energy-resolved photon-counting detector. The choice of tube voltage was based on maximizing the number of detected coherent scattered photons relative to the absorbed dose and Compton-scattered photons while also reducing the effect of Compton scattering in the detector. The 3.5 mm of added aluminum filtration removes photons with low energy (< 30 keV) so the do not reach the skull where they can be absorbed quickly and never reach the detector.

The voxelized human brain phantom consisted of 15 cm diameter of uniformly randomly distributed white and grey matter with a skull contour 5 mm thick with 1-mm voxel size. A spherical region with an embedded *β*A target (uniformly distributed) was digitally inserted inside the brain model in the white and grey matter background. Simulations with different diameters for the spherical region with *β*A were performed. Brain phantoms with and without *β*A were simulated to evaluate the technique’s ability to identify targets and to determine the radiation dose required to obtain an acceptable signal.

### Coherent scattering form factors

A key requirement for the development of the field of medical x-ray scatter imaging is the characterization of the differential scattering cross sections of tissues and phantom materials, given by:

$$\begin{array}{c} \frac{d\sigma_{\text{coh}}}{d\Omega }=\frac{r_{e}^{2}}{2}\left(1+\cos^{2}\left(\theta \right)\right)F_{\text{Mol}}^{2}\left(x,Z\right), \end{array}$$ (1)

where Ω is the solid angle, *r*_*e*_ is the classical electron radius, *θ* is the scattering angle, *Z* is the atomic number, *F*_Mol_(*x*, *Z*) is the coherent scattering molecular form factor describing interference effects between scattering events from electrons in the material and *x* is the momentum transfer defined as:

$$\begin{array}{c} x=\frac{E}{hc}\text{sin}\left(\theta /2\right). \end{array}$$ (2)

The coherent scattering form factors can be obtained from measured x-ray coherent scatter profiles [12–14]. In this study, the coherent scattering form factors of brain tissues were estimated from x-ray coherent scatter profiles measured by Felici *et al*. [15] and Liu *et al*. [16] and their elemental composition materials from the PENELOPE 2006 database [17]. From the x-ray coherent scatter profiles (∝ *dσ*_coh_/*d*Ω), the coherent scattering form factors can be extracted by removing first the polarization factor *p*(*θ*) = (1 + *cos*(*θ*)^2^)/2; therefore the square form factor is obtained in relative terms that need to be scaled to absolute values. It is known from theoretical considerations [18–20] that the measured coherent scattering molecular form factor, $F_{Mol}^{2}\left(x\right)$, asymptotically approaches the independent atom approximation (IAA) form factor $F_{\text{IAA}}^{2}\left(x\right)$ given by:

$$\begin{array}{c} F_{\text{IAA}}^{2}=\sum n_{i}F^{2}\left(x,Z_{i}\right), \end{array}$$ (3)

where *n*_*i*_ is the weight fraction of element *i*, *Z* is the atomic number, *F*(*x*, *Z*) is the coherent scatter form factor for element *i*. As a result, the absolute values of $F_{\text{Mol}}^{2}\left(x\right)$ could be estimated by renormalizing the data to fit the *F*_IAA_ values at sufficiently large momentum transfer values, *x* ranging from 4 to 4.5 nm^−1^.

**Fig 2:**
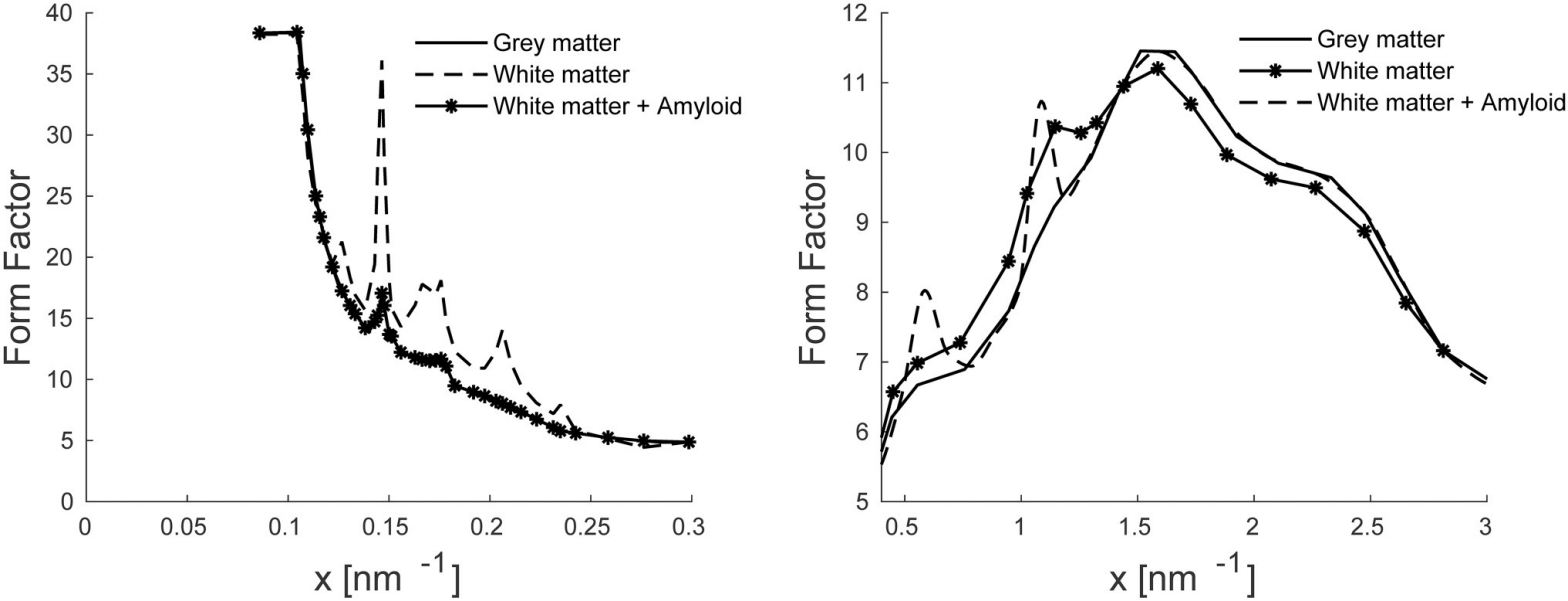
Coherent scattering form factor for grey, white and white plus *β*A materials used in the simulation.

Finally, we assumed that the *β*A region is composed of white matter with modified coherent scattering form factor. We used the molecular form factors of white matter for the region with *β*A with two added peaks as shown in Fig 2. These two peaks were at 0.55 nm^−1^ and 1.064 nm^−1^. The locations, amplitudes and widths of these synthetic peaks were also extracted from measured x-ray coherent scatter profiles of human breast tissues containing plaque [16].

### Detector model

The MC-GPU code assumes use of a flat panel detector with an ideal energy-integrating detector having 100% detection efficiency. To simulate the photon-counting detector of the proposed coherent scatter system, the detector geometry was implemented in a voxelized geometry to account for the effect of scatter inside the detector. An overview of the simulated detector is shown in Fig 1(b). Fluorescence and charge transport effects were not considered in the simulation. We assumed a fully attenuating thin layer between the strips (the detector planes along Z direction). Given these assumptions and for Compton events in the silicon, the remaining photon energy is determined by subtracting the deposited energy. If the photon leaves the first detector plane (strip) it entered, it is assumed to have escaped. The detector image tallies in MC-GPU were modified and the sum of energy deposited per incident photon at each detector element was calculated. Based on the accumulated deposited energy, we added one count to the corresponding energy bin. For this study, we used eight uniformly-distributed energy bins ranging from 30 to 60 keV.

### Data analysis

Data from pencil beam acquisitions are in the form of photon counts across 400×400 pixels and eight energy channels, *I*_*m*_(*m*_*x*_, *m*_*y*_, *E*), where *m*_*x*_ and *m*_*z*_ are the horizontal and vertical pixel numbers and *E* is energy. The amount of data can be reduced by averaging. In this study, the pixel numbers and the energy channels were converted into *x*-values or momentum transfer by factoring in the sample-(focused point of the silicon strips)-to-detector distance.

$$\begin{array}{c} x=\frac{E}{hc}sin\left(\theta \left(m_{x},m_{z}\right)/2\right), \end{array}$$ (4)

where *hc* is the is product of Plank’s constant and the speed of light in a vacuum. Finally, the measured x-ray coherent scatter profile can be written as follows:

$$\begin{array}{c} I_{s}\left(x\right)=\sum_{x=x0}^{xmax}I_{m}\left(x\right)/N\left(x\right), \end{array}$$ (5)

where *N*(*x*) is the number of pixels that record coherently scattered photons with *x* momentum transfer. *N*(*x*) also can take into account energy effects caused by the polychromatic nature of the x-ray source and attenuation through the sample by considering the measured spectrum at 0° (the transmitted beam).

The first step involved considering the difference between the *x*-momentum transfer averaged scatter intensities from a region containing *β*A plaque and a proximal control region with no plaque, $I_{s}^{B}$ and $I_{s}^{C}$, respectively to remove a large part of common materials:

$$\begin{array}{c} \Delta I\left(x\right)=I_{s}^{B}-I_{s}^{C}. \end{array}$$ (6)

The Δ*I*(*x*) spectra should exhibit two diffraction peaks. The intensity of these two peaks reflect both the presence of amyloid plaque in the inspected region and its regional density or concentration. The second step was a more detailed analysis, using intensities and major peak locations to identify the presence of *β*A in the brain.

## Results and discussion

**Fig 3:**
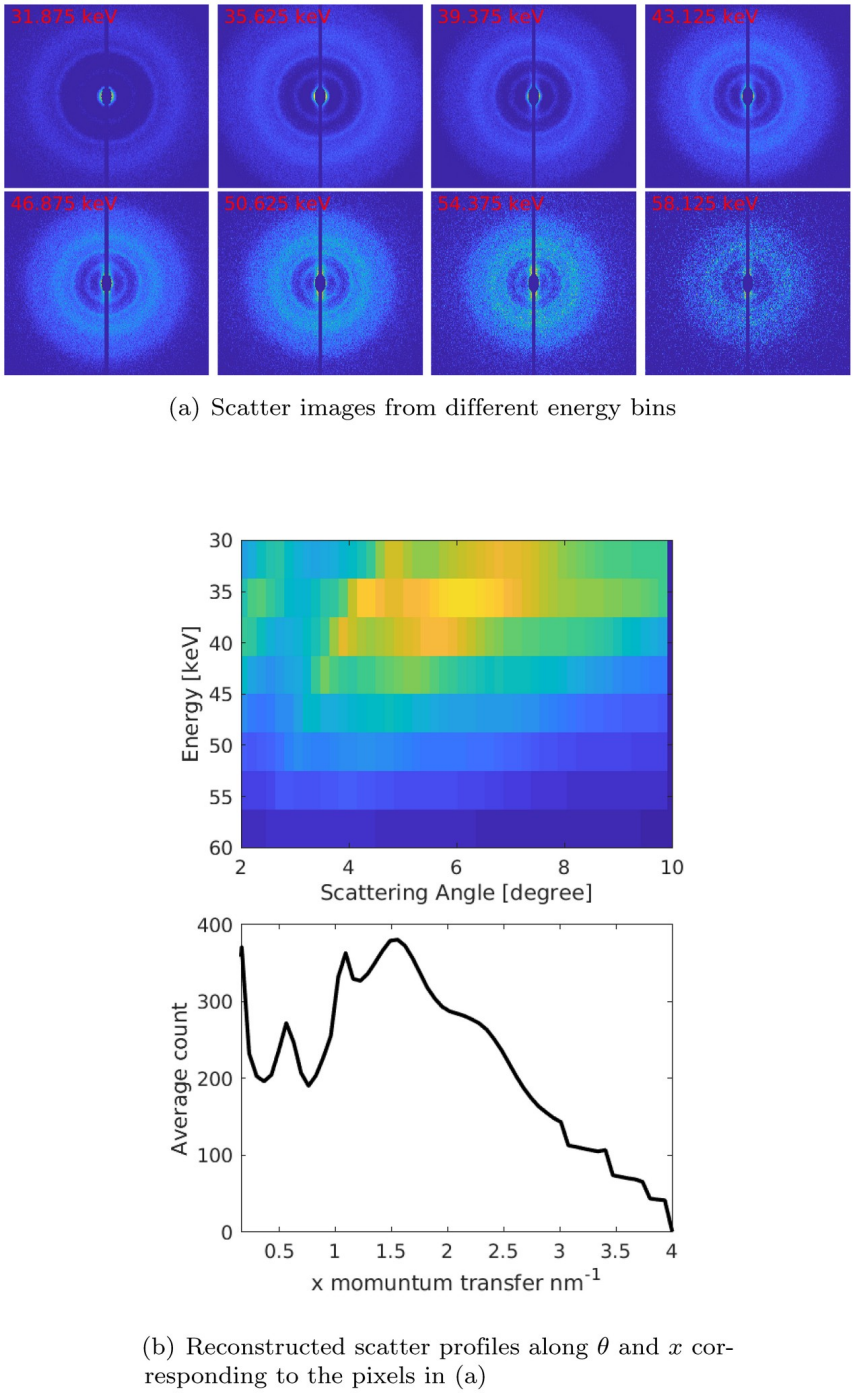
(a) X-ray coherent scatter images acquired from amyloid phantom of 5 mm diameter and x-rays generated at 60 kV. (b) Top row: energy-angle diagram, the *z*-axis shows the number of counts recorded. Bottom row: *x*-calibrated spectrum after converting the energy scale into momentum transfer (*x*) scale as described in the text.

Fig 3 illustrates how energy-resolved scatter images and the a priori knowledge of the scattering angle at each pixel can be used to gain insight into the data reduction process. Consider the case of using 60 keV spectra with eight energy windows evenly distributed from 30 to 60 keV and measuring scatter from a simulated spherical region with *β*A of 5 mm diameter. Fig 3(a) shows eight energy-resolved scatter images and Fig 3(b) shows its corresponding coherent scatter data as a function of energy and angle. The bottom plot shows the calibrated spectrum after converting energy into momentum transfer (*x*) as described in the data analysis section. As can be seen in the energy-angle diagram (Fig 3(b)) for large scattering, the peak energy position appears at lower energies for the same momentum transfer. In addition, the spread in energy is larger at higher energies.

**Fig 4:**
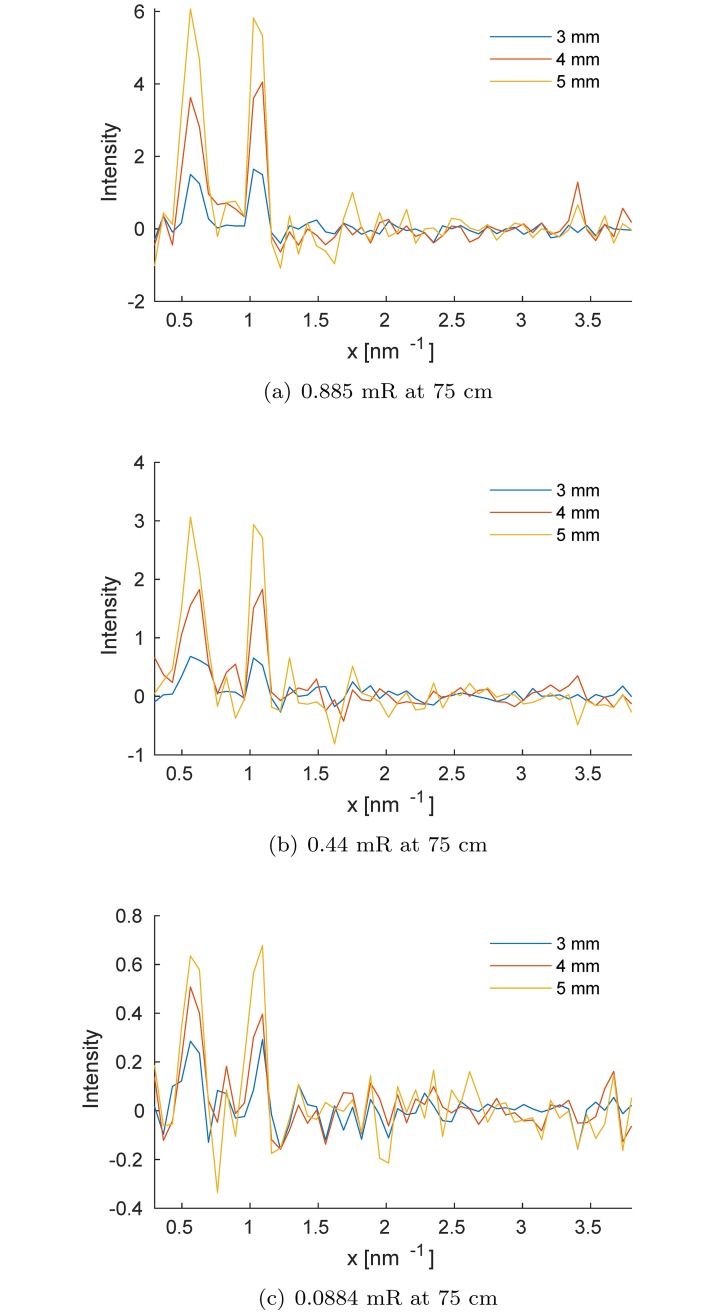
Signal amplitude of the *β*A that corresponds to the difference between the two scatter spectra measured with and without simulated amyloid inserts for three different incident exposure levels and for various diameters of amyloid inside the effective inspected volume.

Fig 4 shows signal contrast curves for varying simulated amyloid targets embedded in 15 cm brain phantom. To explore the effect of x-ray exposure, we show results for 0.088, 0.442 and 0.884 mR measured at 75 cm from the source. As expected, we attained better visualization of the amyloid signature peaks with larger targets. For all amyloid target sizes, results showed that the corresponding diffraction peaks increased with x-ray exposure level.Peaks were observed at 0.55 nm^−1^ and 1.064 nm^−1^ were caused by scattering from the amyloid target. The *β*A diffraction peaks at 0.55 and 1.064 nm^−1^ could be used to detect plaque, because they were visible even with low fraction of *β*A in the inspected brain volume.

**Fig 5:**
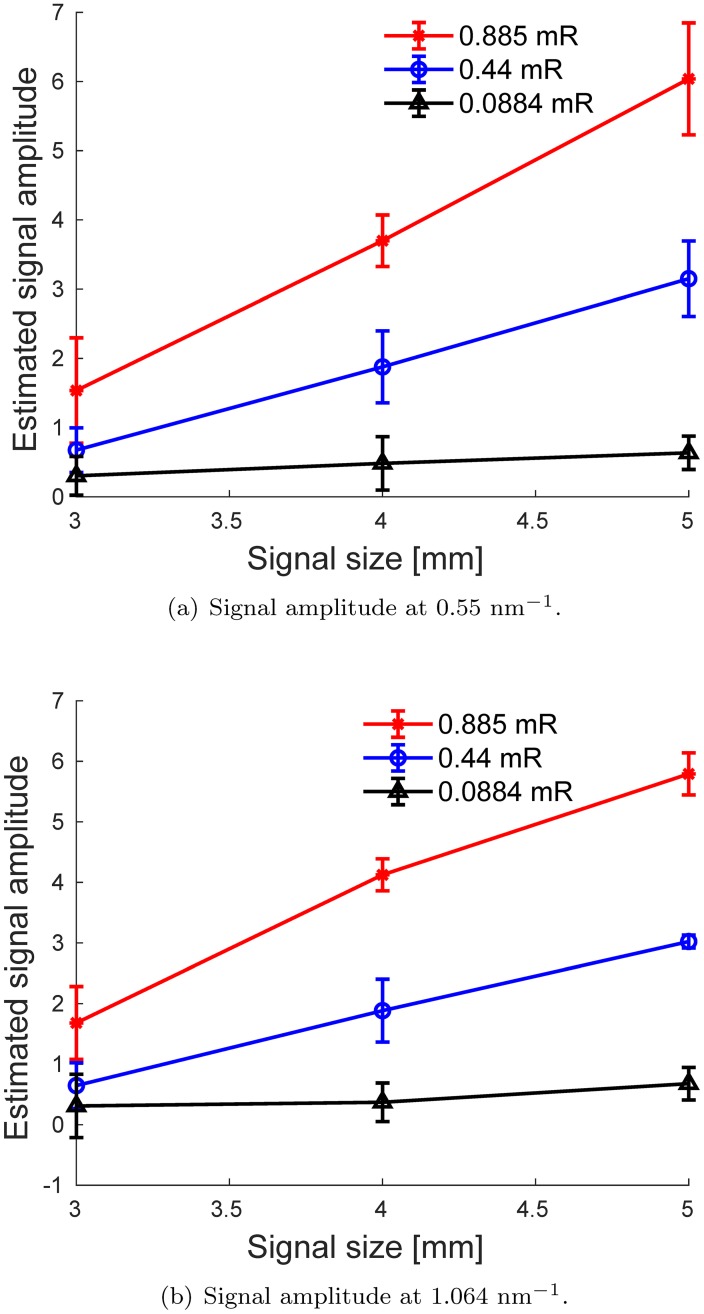
Comparison between the average estimated signal amplitude for the amyloid diffraction peaks shown in Fig 4, for three different exposure levels. The vertical error bars correspond to ±1 standard deviations computed from 10 independent realizations.

Finally, we performed 10 independent simulations for each signal size and exposure level. For each realization, we computed the signal amplitude between the peak intensity *I*_*peak*_ and the background of the Δ*I*(*x*) spectrum, *I*_*back*_ (as the mean of Δ*I*(*x*) at 2 nm^−1^ ≤ *x* ≤ 2.5 nm^−1^), *A* = *I*_*peak*_ − *I*_*back*_. The average of these values and their standard deviations, computed from 10 realizations, versus signal size in the simulated brain phantom for both diffraction peaks, are plotted in Fig 5. As expected, better performance can be achieved with increased exposure. We also observed a linear correlation between signal amplitudes and *β*A signal size. Therefore, with proper calibration, the relative intensities of the peaks could be used to estimate the total mass of the *β*A inside the inspected volume, which could provide useful information for the evaluation of *β*A aggregates in the brain.

Results from this simulation study suggest that it might be feasible to use spectral coherent scatter imaging for *β*A identification in the brain. It had several limitations, however. Some of the detector physics that might have affected the results, including charge collection deficiency, pulse pile-up, charge sharing, and fluorescence effects in the silicon strips, were not modeled in our study.

Additionally, as discussed previously, the absolute scatter intensity of brain tissues used in the study was based on an experimental study by Liu *et al*. [16] and may significantly vary among subjects. This variability is not accounted for in this study. Further statistical analysis of a large number of number of brain tissue samples containing *β*A is necessary for investigating the sensitivity and specificity of this approach, as well as realizing an experimental physical demonstration of the imaging system.

## Conclusion

We tested the use of a spectral x-ray coherent scatter system with a silicon strip detector for brain *β*A detection and quantification. The study was carried out by simulating a multiple-angle, energy-dispersive coherent scatter system with a pencil beam configuration, considering a polychromatic x-ray source and an energy-resolving silicon strip array with edge-on geometry. The results suggest that the proposed x-ray diffraction system enables the use of high energy x-ray spectra and therefore has the potential to be used for accurate in vivo detection and quantification of brain *β*A with acceptable levels of radiation exposure. This study provides encouraging first results in evaluating the feasibility of the proposed *β*A identification methodology. Future work should investigate identification performance in more relevant clinical conditions using a more realistic detector model and a broad range of brain samples with nonuniform backgrounds containing *β*A targets.
